# Statistical analysis plan for cluster randomised trial to evaluate a community-level complementary food safety and hygiene and nutrition intervention in Mali: the MaaCiwara study

**DOI:** 10.1186/s13063-024-08328-x

**Published:** 2024-07-16

**Authors:** Laura Quinn, James Martin, Evans Asamane, Semira Manaseki-Holland, Richard J. Lilford, Lacina Traore, Jacqueline Thompson, Samuel I. Watson, Karla Hemming

**Affiliations:** Institute of Applied Health Research, University of Birmingham, Birmingham, UK

**Keywords:** Statistical analysis plan, Cluster randomised controlled trial, Food safety and hygiene

## Abstract

**Background:**

Diarrheal disease is a significant cause of morbidity and mortality in under-fives in many low- and middle-income countries. Changes in food safety, hygiene practices, and nutrition around the weaning period may reduce the risk of disease and improve infant development. The MaaCiwara study aims to evaluate the effectiveness of a community-based educational intervention designed to improve food safety and hygiene behaviours, as well as child nutrition. This update article describes the statistical analysis plan for the MaaCiwara study in detail.

**Methods and design:**

The MaaCiwara study is a parallel group, two-arm, superiority cluster randomised controlled trial with baseline measures, involving 120 clusters of rural and urban communities. These clusters are randomised to either receive the community-based behaviour change intervention or to the control group. The study participants will be mother–child pairs, with children aged between 6 and 36 months. Data collection involves a day of observation and interviews with each participating mother–child pair, conducted at baseline, 4 months, and 15 months post-intervention.

The primary analysis aims to estimate the effectiveness of the intervention on changes to complementary food safety and preparation behaviours, food and water contamination, and diarrhoea. The primary outcomes will be analysed generalised linear mixed models, at individual level, accounting for clusters and rural/urban status to estimate the difference in outcomes between the intervention and control groups. Secondary outcomes include maternal autonomy, enteric infection, nutrition, child anthropometry, and development scores. In addition, structural equation analysis will be conducted to examine the causal relationships between the different outcomes.

**Trial registration:**

International Standard Randomised Controlled Trial Number (ISRCTN) register: ISRCTN14390796. Registered on 13 December 2021.

**Supplementary Information:**

The online version contains supplementary material available at 10.1186/s13063-024-08328-x.

## Background

Diarrheal disease is a significant cause of morbidity and mortality in under-fives in many low- and middle-income countries [1]. Approximately 525,000 children under 5 die each year from diarrhoeal disease, meaning it is the second highest cause of death for under-fives worldwide [2]. Apart from mortality, diarrhoeal disease also leads to malnutrition and developmental delays [3, 4]. Changes to food safety, hygiene practices, and nutrition around the weaning period may reduce the risk of disease and improve infant development [5].

The objective of the MaaCiwara study is to evaluate the effectiveness to evaluate the effectiveness of a multi-faceted community-based educational intervention that aims to improve food safety and hygiene behaviours and enhance child nutrition. This will be done using a cluster randomised controlled trial with baseline measures, with 120 clusters comprising of rural and urban communities randomised to either the community-based behaviour change intervention or control group. Further details on the background and rationale for the study are outlined in more detail in the protocol [6].

In this article, we describe the detailed statistical analysis plan (SAP) for the MaaCiwara study, which describes the planned analyses and presentation of results for this study. Any subsequent analysis will have a more exploratory nature, though they will follow the general layout of the strategy here. The statistical analysis plan follows the guidelines for the content of SAPs in clinical trials; details are reported in Supplementary material A [7]. Names, affiliations, and roles of SAP contributors are presented in Supplementary material B. The final report of findings will follow the extension of the Consolidated Standards of Reporting Trials (CONSORT) 2010 guidelines for reporting cluster randomised trials [8].

## Objectives

The MaaCiwara study objectives were published in the trial protocol [6]. Here, we break down these objectives into specific aims that are covered in this statistical analysis plan. For simplicity, we use the standard terms of primary outcomes, secondary outcomes, and implementation outcomes (all outcomes are listed in tabulated format). Additional aims, such as those related to estimating the cost-effectiveness of the intervention, will be described elsewhere.

### Primary aim

- ▪ Evaluate the effect of the intervention on primary outcomes at 15 months post-intervention

### Secondary aims

- ▪ Evaluate effect of intervention on primary outcomes at 4 months post-intervention
- ▪ Summarise descriptively the implementation outcomes at both 4 and 15 months post-intervention
- ▪ Evaluate the effect of the intervention on secondary outcomes at both 4 and 15 months post-intervention
- ▪ Evaluate the effect of the intervention on primary and secondary outcomes separately for urban and rural settings at both at 4 and 15 months post-intervention

### Additional objectives

- ▪ Explore mediation pathways that affect the effect, or lack thereof, of the intervention on key primary outcomes, as mediated by other outcomes (including outcomes measured at 4 months)

## Study methods

### Trial design

The MaaCiwara study is a mixed-methods, parallel group, two-arm, superiority cluster randomised trial with baseline measures to evaluate a community-level complementary food safety and hygiene and nutrition intervention in Mali. Observations are collected (using repeated cross-sectional sampling) at baseline, mid-line (4 months post-intervention roll-out, post control condition roll-out in the control clusters), and end-line (15 months post-intervention roll-out).

### Cluster recruitment

Clusters (*N* = 120) are urban and rural communities, recruited in equal numbers. Clusters were recruited based on the following criteria. Urban clusters were created in Bamako city, designed to include predominantly poor communities with 500 to 2000 people and as much space between clusters as possible. Rural clusters were villages in the Bamako, Sikasso, and Sego triangle region, with a requirement that they were a minimum of 5 km apart. Eligible clusters were stratified by urban/rural and population size (≤ / > median) and the availability of (Community Lead Total Sanitation) CLTS, for rural clusters only. Clusters were randomly ordered within strata and approached for inclusion in that order.

### Trial interventions

#### Intervention

The intervention is an adapted version of a community level complementary-food hygiene and safety intervention previously evaluated in the Gambia, the MaaChampion Gambian study [9] and will include adaptions from the findings of a mixed-methods formative research study conducted in seven rural and urban communities in Mali.

The format of the intervention will be described elsewhere, but in brief, the intervention will consist of four days of campaign community visits dispersed across 28–35 days. Implementation will be through intervention teams including community leaders and members. The full intervention will be described in a separate publication in compliance with the Template for Intervention Description and Replication (TIDieR) checklist [10].

#### Control

The plan was for the control clusters to receive a 1-day community-based campaign on the use of water in homes with content similar to intervention but not containing equivalent content on food and water preparation, hygiene, child nutrition, or hygienic play. However, this was later changed to a 1-day community-based campaign on vaccination during COVID. The control was implemented at around the same calendar time as the intervention was rolled out.

### Blinding

Due to the nature of the intervention, blinding of mothers and the intervention team will not be possible. However, mothers will be aware there is an intervention or control group programme but not be informed individually the rationale for the trial as permissions for cluster participation will be taken from community leaders. The assessment team is delinked from the trial team; hence, there is no mention of the trial during the assessments. At the assessments, mothers will be informed that the assessment is investigating how children aged 6 to 24 months and their mothers spend their days in rural and urban Mali and to support delivery of local health and social services. Therefore, data collectors will be trained for, and mothers consented to, the conduct of a larger assessment of household’s food and water consumption, health, and childcare and behaviour. New independent field teams will be recruited for the 15 months outcome assessments and will not be informed of the intervention or the inter-village comparison. The statisticians completing the analysis will be blinded as to treatment allocation.

### Randomisation

Randomisation to the intervention or control group was stratified by rural and urban status using random block sizes of two and four to maintain both balance within stratum and prevent predictability of assignment (implemented in Stata v17.0). Randomisation was implemented after baseline data collection in all clusters and concealed until immediately prior to intervention roll-out. The plan was for an independent statistician to generate an allocation sequence. Due to staff turnover, this statistician who at the time of randomising was independent and then became part of the study team at a later date.

### Timing of outcome assessments

All outcomes are measured at baseline, mid-line (around 4 months post-intervention roll-out), and end-line (around 15 months post-intervention roll-out). The windows of data collection extended 3 to 4 months to allow for practicalities of implementing the data collection across multiple clusters. The plan was for all three rounds of data collection took place at the same time of year (February to June or early July) with Ramadan month as a break in the mid-data collection phases.

### Primary outcome measure

There are three primary outcomes, listed in Table 1. Note that the full detailed description of these outcomes is available in the study protocol, and a brief description is provided here.

**Table 1:** Primary outcomes descriptions (all measured at baseline and 4- and 15-month post measurement with primary assessment time at 15 months)

**Outcome category**	**Description**	**Data collection method**	**Units**
**Primary outcomes**			
Water and food safety and hygiene behaviour	The number of times the behaviour is observed out of all opportunities (includes (1) washing caregiver/mother hands with soap before feeding child, (2) washing child’s hands with soap before child feeding, (3) heating the food for the child if stored > 3 h or bought as street food, (4) boiling the drinking water of the child no longer than 24 h before giving to child)	Observation (number not fixed)	Number of opportunities met (binomial)
Food and water contamination	*E. coli* count in child’s food and water samples	Sample collection and field testing	Colony-forming units/gramme (cfu/g) (count)
Diarrhoea	Observation of stool consistency (watery diarrhoea) by field data collectors and history of at least 2 other such stools in the last 24 h	Observation of collected stool by field worker	Dichotomous

### Secondary outcome measures

The secondary outcomes are listed in Table 2. They are split into three groups, knowledge and behaviour, short-term microbiological and clinical outcomes, and long-term physiological outcomes. All secondary outcomes are measured at all three time points. Details of additional secondary outcomes are given in Supplementary material C. The implementation outcomes are listed in Table 3 (these are measured only in the intervention clusters and only at 4 and 15 months).

**Table 2:** Secondary outcome descriptions (all measured at baseline* and 4- and 15-month post measurement with primary assessment time at 15 months)

**Outcome category**	**Description**	**Data collection method**	**Units**
***Knowledge and behaviour***			
Nutrition	Minimum acceptable diet based on minimum dietary diversity and minimum meal frequency they are fed during the day (DHS survey, see below) Minimum dietary diversity: at least five out of eight key requirements which consist of breastmilk; grains, roots and tubers; legumes and nuts; dairy products (infant formula, milk, yogurt, cheese); flesh foods (meat, fish, poultry and liver/organ meats); eggs; vitamin A rich fruits and vegetables; other fruits and vegetables Minimum meal frequency: score 2 or more solid or semi-solid or soft feeds for breastfeeding children age 6–8 months, or 3 or more solid or semi-solid or soft feeds for breastfeeding children age 9–23 months; or 4 or more solid or semi-solid or soft or milk feeds for non-breastfeeding children age 6–23 months where at least one of the feeds must be a solid, semi-solid, or soft feed	Survey	Dichotomous (composite)
Geophagy	Number of behaviours observed from total opportunities in assessment of child play environment and behaviour over the observation period. Count of events: 1. Observed no geophagy, 2. Observed supervision during play/sitting, 3. Observed sitting/playing on a clean surface. Total opportunities: where child is observed every 30 min when not sleeping or otherwise occupied.	Observation (number not fixed)	Number of opportunities met (binomial)
Maternal autonomy	Women’s autonomy measure (from DHS survey). Proportion (of *n* = 9) of household decisions the woman participates in.	Survey	Number of opportunities met (binomial)
***Short-term microbiological and clinical outcomes***			
Acute respiratory infection	Parental report of cough and difficulty breathing in past 7 days	Survey	Dichotomous
Diarrhoea hospitalisation	In-patient hospitalisation (if given a bed to stay for observation, tests or treatment for > 3 h) for diarrhoeal disease in the past 3 months	Survey	Dichotomous
Enteric infection	Qualitative PCR of the following pathogens: ETEC; EAEC; EPEC; Astrovirus; Sapovirus; Rotavirus; Adenovirus; Giardia; Cryptosporidium; *E. histolytica*	Sample collection and lab testing	Dichotomous (any pathogen)
**Long-term physiological outcomes**			
Physical growth	Weight (take average of three measures taken at each point in time)	On-site measurement	Weight for age (*z*-score [WHO International Growth Tables])
Physical growth	Height (take average of three measures taken at each point in time)	On-site measurement	Height for age (*z*-score [WHO International Growth Tables])
Cognitive development	ASQ3 score for age^a^	Survey/observation	Count

^a^ASQ3 was not measured in the baseline survey

**Table 3:** Implementation outcome descriptions (all measured and 4- and 15-month post measurement only in the intervention clusters)

**Outcome category**	**Description**	**Data collection method**	**Units**
Fidelity (coverage)	Number of visits of the intervention delivered as planned	Investigator report	Count
Uptake	Number of mothers who reported the intervention as a source of knowledge for food hygiene/safety or optimal nutrition	Survey	Count

### Sample size

The sample size justification was reported in full in the published protocol [6]. The design used stratified randomisation to allocate 120 communities to two arms and recruit 3240 mother–child pairs (27 per cluster-period) in total, equally distributed across baseline, mid-line (4 months post-intervention), and end-line (15 months post-intervention, primary assessment time) as three cross-sectional data collection rounds. The mid-line data is not included in the power calculations since it does not contribute to the primary outcome analysis (primary assessment time is 15 months, but conditioning on baseline data). For the three primary outcomes, we will collect:

1. Water and food safety and hygiene behaviour observations: a binomial outcome with an anticipated four observations per pair for all 27 mother–child pairs
2. Food and water *E. coli* contamination: a count outcome from samples randomly selected from the 27 total pairs (assumed to be 10 samples for the purpose of the sample size calculation)
3. Diarrhoea: a single dichotomous observation for each of the 27 mother–child pairs (1 per pair)

We report the power for the three primary outcomes for a range of target effect sizes assuming an intraclass correlation coefficient (ICC) of 0.02, a cluster autocorrelation coefficient (CAC) of 0.8, and a 5% significance level in Supplementary material D1. We also consider a range of plausible values of the ICC and CAC, and power calculations with the alternative values are provided in Supplementary materials D2 and D3. For example, for the first of the primary outcomes, water and food safety and hygiene behaviour, assuming this appropriate practice is 50% under the control condition, we would have 69% power to detect an increase to 55% assuming an ICC of 0.02 and a CAC of 0.8.

Following recommended guidance [11], we have used values for ICCs and CACs informed by similar outcomes in similar settings (noting that ICCs for process outcomes tend to be higher for those for clinical outcomes [12]). For the CAC, where we have limited information on values, we have used the values of 0.8 and 0.9 as recommended in the literature [13].

We did not include planned covariate adjustment nor the randomisation procedure in the power calculations. Whilst for two of the primary outcomes there will be multiple measures per mother–child pair, we only assumed one in our power calculations. Thus, we anticipate that power calculations are likely to be conservative all other assumptions holding. Since the outcomes will be evaluated using repeat cross-sectional rounds, loss-to-follow-up of specific individuals is unlikely to be an issue. Variation in cluster sizes was not allowed for as 27 mother–child pairs will be recruited in each cluster period.

The sample size calculation for the primary outcomes was implemented using an RShiny app for cluster trials https://clusterrcts.shinyapps.io/rshinyapp/ with methodology described elsewhere Hemming et al. [14]. Power for the subgroups presented below was computed by determining the power for an interaction effect for a logistic regression model, calculated using the approach described by Demidenko (2010) and inflated using a design effect approach.

### Interim analyses and stopping guidance

We will not specify any “stopping rules” for the trial as the intervention is community-based, non-invasive, and non-clinical and is very unlikely to present any risk of harm to the study participants.

### Timing of final analysis

The final analysis for the trial will occur after all the clusters have been randomised; the baseline, mid-line, and end-line data has been collected; the data has entered onto the trial database and the data has been validated as being ready for analysis.

### Data quality monitoring

At baseline, mid-line, and end-line data collection points, data quality will be monitored. To this end, all covariates and outcomes will be summarised descriptively, stratified by treatment arm, with information on the number of missing data. This analysis will be undertaken blind to the treatment arm.

### Trial comparisons

All comparisons referred to in this document are between the community intervention group and the standard care control group.

## Statistical principles

### Confidence intervals and *p*-values

All estimates of differences between the intervention and control group will be reported with 95% confidence intervals and *p*-values associated with a two-sided test at the 5% significance level.

### Adjustments for multiplicity

There will be no correction for multiple testing as each of the three primary outcomes measure different domains and our articulated interpretation plan intends to consider a narrative based on findings for all three outcomes concurrently and not independently. Thus, “success” of the intervention will not be defined by statistical significance testing alone.

### Analysis populations

All analyses will be completed using an intention-to-treat (ITT) approach. Participants and clusters will be analysed in the group to which they were allocated.

### Protocol deviations

Any deviations from this SAP will be described and the reasoning behind the deviations will also be given (as shown in Supplementary material E).

## Trial population

### Recruitment

A CONSORT flow diagram will be produced to describe both the flow of clusters and participants throughout each stage of the trial (see Supplementary material F1). For clusters, this information will include the number of clusters recruited, randomly allocated to intervention or control groups, lost to follow-up, and number analysed for primary outcomes. For participants, this information will include the number of mother–child pairs recruited and the number of mother–child pairs analysed for the primary outcomes at each cluster period.

### Baseline characteristics

The study population characteristics will be summarised at cluster level and individual level (listed in Supplementary material F2). Characteristics will be summarised by intervention and control group and stratified by time period (baseline and 4 months and 15 months post-intervention). Categorical data will be summarised by number and percentages. Continuous data will be summarised by means and standard deviations, or medians and interquartile ranges, as appropriate.

## Analysis methods

The main aim of this study is to evaluate the effectiveness of the intervention to promote improved drinking water and complementary food safety and hygiene behaviour to reduce water and food contamination and diarrhoea.

This will be done by estimating the difference in outcomes between the intervention and control group using generalised linear mixed models, accounting for clustering. Further details on the analysis methods for the primary outcomes, secondary outcomes, and additional analysis are given in the following sections.

A template for reporting results for baseline characteristics, primary and secondary outcomes, and additional analysis are given in Supplementary material F2-F6. For any composite outcomes, we will also report effects on components of composites as exploratory analyses.

### Primary outcome analysis

All outcomes will be summarised by intervention and control group. For continuous outcomes, we will summarise using means and standard deviations, or medians and inter-quartiles ranges, as appropriate. For categorical outcomes, we will summarise using number and percentages. Treatment effects for all three primary outcomes will be estimated at both 4 months and 15 months post-intervention (the primary assessment time is 15 months). Model approaches are described below and consist of separate models to estimate the effect at 4 and 15 months (both adjusting for baseline values). Because we do not use one joint model, we will not be able to directly compare treatment effects across the two assessment points (4 and 15 months).

For all outcomes, a generalised linear mixed model will be fit, and the level of observation is an individual nested within cluster-periods. Data analysis will be undertaken in long format with variables: cluster, outcome, time period indicator (0 months—coded 0, 4 months—coded 1 in the 4-month analysis, 15 months—coded 1 in the 15-month analysis), and study arm (0 control, 1 intervention). Each model will include an intercept, an indicator for whether the cluster had the intervention at the time (an interaction between study arm and time period), and a post-intervention time period indicator (time period). The covariate used in the randomisation (urban or rural status) will be included in each model. To account for the clustered nature of the data, random effects will be included for cluster and cluster-periods (observation within one cluster during one time-period).

For continuous outcomes, we will use a multilevel mixed-effects linear model with restricted maximum likelihood (REML) to estimate a mean difference.

For dichotomous outcomes, we will use a generalised linear mixed model with a binomial distribution and logit link to estimate odds ratios with cluster-robust standard errors. The absolute difference will be estimated averaging over the study population (the average marginal effect) using a marginal standardisation approach and will be reported with cluster-robust standard errors.

For binomial outcomes, we will use a generalised linear mixed model with a binomial distribution and logit link to estimate odds ratios. For example, the primary outcome of water, food safety and hygiene behaviours, the numerator is the number of events where the mother practiced a pre-specified behaviour, and the denominator is the number of possible opportunities to practice those behaviour. The model accounts for the mothers having different numbers of opportunities. The absolute differences will be calculated using the same marginal standardisation approach as for binary outcomes, together with cluster-robust standard errors. The use of the cluster-robust standard errors will allow for over dispersion due to the correlation between the multiple opportunities within mothers as well as mothers within clusters.

For count outcomes, we will use a generalised linear mixed model with a Poisson distribution and log link to estimate rate ratios and again with cluster-robust standard errors. The use of the cluster-robust standard errors will protect against overdispersion in the count outcome. The absolute differences will also be calculated using the same marginal standardisation approach as for binary outcomes. For the primary outcome count of food and water contamination, if this variable is highly skewed, we will consider transformations in the first instance, but should no transformation be appropriate, this outcome will be dichotomised.

For implementation outcomes, which are only measured in the intervention clusters, we will report descriptive statistics only as no comparison will be possible between intervention and control clusters.

For generalised linear mixed models, the maximisation method will be the Newton–Raphson method and the integration method will be the mean–variance adaptive Gauss-Hermite quadrature, which is the Stata default.

All estimates will be reported with 95% confidence intervals and *p*-values associated with a two-sided test of no difference. We will also report ICCs with 95% confidence intervals for binary and continuous outcomes on the latent scale.

The model assumes a cross-sectional sampling structure within cluster periods. If there are a high proportion of mothers appearing in multiple rounds (with the same or different children), and we can successfully link observations, then we will treat the outcomes as repeated measures and include a mother-level random effect term. No small sample correction will be included for the primary outcome analysis as the number of clusters is sufficiently large to maintain the type I error rate [15].

A statistician will complete the primary analysis independently in duplicate. Any discrepancies will be resolved by comparison of code and a third person if necessary.

### Primary outcome analysis—model non-convergence

If the generalised linear mixed models with random effects for clusters and cluster periods do not converge, the following approaches will be performed in this order. First, the cluster by period random effect will be removed, and generalised linear mixed models will be fit with only a random effect for cluster. If the models still do not converge, a weighted cluster level analysis will be completed. For each cluster in each time period, we will calculate a cluster-level summary statistics, and models will be fit with a sampling weight representing the number of mother–child pairs in the cluster during that period. Under both mitigation approaches, we will use cluster-robust standard errors to protect against misspecification of the correlations.

### Covariate-adjusted analysis

For the primary outcomes, the analysis is not adjusted for any individual-level covariates (but will adjust for the covariates used in the randomisation, as per the description above). Two additional covariate adjusted analyses will be completed for the primary outcomes at both 4 months and 15 months post-intervention. Firstly, an analysis adjusting for the following cluster covariates: number of children 6–24 months (as an indicator of the cluster population size (< / ≥ median)); presence of school; presence of community-led total sanitation (CLTS) and whether there is a health centre located in the cluster. Secondly, an analysis adjusting for the individual covariates will be completed. For each outcome, there are a slightly different set of covariates for the adjustment. All primary and secondary outcomes will adjust for the following covariates: age of the child (continuous), mother’s educational status (categorical), working mother at the time of the interview (categorical), and number of children < 5 years in the household (categorical) with the exception of the following two outcomes: maternal autonomy, which will adjust for mother’s educational status (categorical), working mother at the time of the interview (binary), marital status—2nd wife (categorical), and age of mother (continuous), and the outcome nutrition, which will adjust for age of the child (continuous), mother’s educational status (categorical), working mother at the time of the interview, and recent breastfeeding initiative (cluster-level variable, categorical). For any continuous covariates, we will explore the need to adjust for non-linear effects using fractional polynomials (continuous covariates will be included in their continuous format and will not be categorised).

### Secondary outcomes

The secondary outcomes are split into three groups: knowledge and behaviour, short-term microbiological and clinical outcomes, and long-term physiological outcomes.

All secondary knowledge and behaviour, short-term microbiological and clinical outcomes, and long-term physiological outcomes will be summarised by intervention and control group. We will estimate the overall treatment effects for the secondary outcomes at 4 months and 15 months post-intervention using the same statistical approach as for the primary outcomes. Both unadjusted and adjusted models will be fitted. Exploratory subgroup analysis will be performed for the secondary outcomes for urban and rural clusters (details below).

### Subgroup analysis

We will conduct an exploratory subgroup analysis to compare the effect of the intervention in urban and rural settings at 4 and 15 months post-intervention (primary assessment time). We will add an interaction between an indicator for urban or rural clusters and the treatment effect. Estimated effect sizes in both relative and absolute terms for urban and rural clusters will be reported along with 95% confidence intervals. In addition, we will report a measure of difference between subgroup-specific treatment effects with 95% confidence intervals. For relative measures, we will calculate the ‘ratio of ratios’, and for absolute measures, we will calculate ‘difference of differences’ using the post-estimation commands. No small sample correction will be included for the subgroup analysis as the number of clusters is sufficiently large to maintain the type I error rate [15].

### Implementation outcomes

All implementation outcomes will be described descriptively only, and there will be no comparison across study arms. To this end, the implementation outcomes will be summarised for the intervention group by counts and percentages at both 4 and 15 months post-intervention and by rural and urban clusters.

### Mediation analysis

The final aim outlined under the objectives is to explore mediation pathways that affect the effect, or lack thereof, of the intervention on key primary outcomes, as mediated by other outcomes (including outcomes measured at 4 months).

To this end, we will fit a series of structural equation models based on the assumed causal relations specified to facilitate interpretation of the mediation pathways of the intervention. Analysis will be split into two main parts, the first part including the primary outcomes (measured at 4 and 15 months) and the second part including both the primary and secondary outcomes (measured at both 4 and 15 months).

For the first part of the analysis, a structural equation model including the intervention and primary outcomes will be fit (see Supplementary material F6 for directed acyclic graphs (DAGs) and tables). A random effect for village will be included to account for clustering. Given the complexity of model fit, and the anticipated infrequency of repeated measures on the same women, we will not include an individual level random effect. Direct, indirect, and total effects will be reported with 95% confidence intervals. The proportion of the total effect between the intervention and the outcome diarrhoea mediated by water, food safety, and hygiene behaviours and food and water contamination will be calculated. This model will also be fit separately for rural and urban clusters as it is expected the effect of the intervention may differ.

For the second part of the analysis, a structural equation model will be fit including the intervention and both the primary and secondary outcomes (see Supplementary material F7 for DAGs and tables). A random effect for village will be included. Direct and indirect effects will be reported with 95% confidence intervals. Estimates of hypothesised casual relationships will also be examined by statistical significance and direction (i.e. positive or negative) of associations.

For each of the models, the fit of the model will be examined by using the standardised root mean residual (SRMR), the root mean square error of approximation (RMSEA), the comparative fit index (CFI), and the Tucker-Lewis Index (TLI). A ‘good’ fit for a model is defined as a SRMR ≤ 0.08, RMSEA ≤ 0.07, CFI ≥ 0.95, and TLI ≥ 0.95 [16, 17]. All analyses will account for all mediator-outcome confounders variables, and these will be those outlined in the covariate adjustment section.

### Distributional assumptions and outlying responses

For the continuous outcomes, distributional assumptions will be assessed. In the first instance, the proposed primary method of estimation in this analysis plan will be followed. If responses are particularly skewed and/or distributional assumptions violated, the impact of this will be examined through sensitivity analysis; this will consist of transformation of responses prior to analysis (e.g. log transformation) in the first instance. For outlying responses, any extreme values will be investigated and if considered to be affecting the integrity of the analysis will be removed for a sensitivity analysis.

### Handling missing data

For the primary outcomes, the analysis is not adjusted for any covariates so there will be no missing covariate data; however, there could be missing outcome data.

For the primary analysis and the additional covariate adjusted analyses, if there is more than 5% missing data, across both the outcome data or covariates, a sensitivity analysis with multiple imputation using chained equations will be performed [18, 19]. All covariates with missing data and the outcome data will be included in the imputation process. Auxiliary variables will be considered for the imputation process. The number of imputations performed will be based on the proportion of missing data in the covariates used. Clustering will be allowed for in the imputation process by using the Realcom Imputation software which allows for random effects to be included.

For the additional analysis, structural equation modelling will be conducted using an available case analysis. Any secondary outcomes with more than 10% missing data will not be included, to maximise the amount of data available.

### Safety data

The intervention is a community-based, non-invasive, and non-clinical and presents minimal risk of harm to the study participants.

## Statistical software

All analysis will be completed in Stata v18 unless otherwise stated. The multiple imputation will be completed using the Realcom Imputation software (as Stata does not allow for random effects as part of its multiple imputation package).

## Trial status

This document represents the most recent version of the SAP (v2.5) based on latest version of the protocol (v1.4). Amendments to the SAP are reported in Supplementary material G. Data collection for the post-intervention period is ongoing, and the anticipated date for completion of follow-up on the last participant is 31 December 2024.

### Supplementary Information

Supplementary Material 1: Supplementary material A. Statistical Analysis Plan (SAP) Checklist v 1.0 2019. Supplementary material B. Names, affiliations, and roles of SAP contributors. Supplementary material C. List of additional secondary outcomes as outlined in protocol. These were termed “alternative outcomes” in the protocol. For each of the outcomes, the difference between the intervention and control group at 4 months post-intervention and 15 months post-intervention (primary assessment time) will be calculated. Supplementary material D1. Power for outcomes in primary outcomes for combination of effect sizes and assuming an ICC of 0.02, a CAC of 0.8, and a type I error rate of 0.05. Power for non-continuous outcomes calculated using a normal approximation. pp = percentage point. For full details see main text. Of note the values in yellow are corrected from those published in the protocol. Supplementary material D2. Power for the three main outcomes for different combinations of effect sizes and assuming an ICC of 0.05, a CAC of 0.8, and a type I error rate of 0.05. Power for non-linear models calculated using a normal approximation. Supplementary material D3. Power for the three main outcomes for different combinations of effect sizes and assuming an ICC of 0.05, an CAC of 0.5, and a type I error rate of 0.05. Power for non-linear models calculated using a normal approximation. Supplementary material E. Deviations from SAP. Supplementary material F1. CONSORT flow diagram. Supplementary material F2. Characteristics of clusters and mothers by time period and intervention allocation. Supplementary material F3. Primary outcome results. Values are number and percentages unless stated otherwise. Supplementary material F4. Subgroup analysis results. Subgroup is location (rural/urban setting). Values are number and percentages unless stated otherwise. Supplementary material F5. Secondary outcome results. Values are number and percentages unless stated otherwise. Supplementary material F6. Additional analysis results, part 1. Supplementary material F7. Additional analysis results, part 2. Supplementary material G. Statistical Analysis Plan (SAP) Amendments.

## Data Availability

Data sharing is not applicable to this article (a statistical analysis plan) as no datasets will be generated or analysed during this stage of the study.

## Abbreviations

ISCRTN: International Standard Randomised Controlled Trial Number
SAP: Statistical analysis plan
CONSORT: Consolidated Standards of Reporting Trials
TIDieR: Template for Intervention Description and Replication
ICC: Intraclass correlation coefficient
CAC: Cluster autocorrelation coefficient
ITT: Intention to treat
