# Supplementary material for: Statistical analysis plan for cluster randomised trial to evaluate a community-level complementary food safety and hygiene and nutrition intervention in Mali: the MaaCiwara study
Source: Trials. 2024 Jul 16;25:483. doi: 10.1186/s13063-024-08328-x (PMC11253307; doi:10.1186/s13063-024-08328-x)
Supplement: Supplementary file 1 — Supplementary Material 1: Supplementary material A. Statistical Analysis Plan (SAP) Checklist v 1.0 2019. Supplementary material B. Names, affiliations, and roles of SAP contributors. Supplementary material C. List of additional secondary outcomes as outlined in protocol. These were termed “alternative outcomes” in the protocol. For each of the outcomes, the difference between the intervention and control group at 4 months post-intervention and 15 months post-intervention (primary assessment time) will be calculated. Supplementary material D1. Power for outcomes in primary outcomes for combination of effect sizes and assuming an ICC of 0.02, a CAC of 0.8, and a type I error rate of 0.05. Power for non-continuous outcomes calculated using a normal approximation. pp = percentage point. For full details see main text. Of note the values in yellow are corrected from those published in the protocol. Supplementary material D2. Power for the three main outcomes for different combinations of effect sizes and assuming an ICC of 0.05, a CAC of 0.8, and a type I error rate of 0.05. Power for non-linear models calculated using a normal approximation. Supplementary material D3. Power for the three main outcomes for different combinations of effect sizes and assuming an ICC of 0.05, an CAC of 0.5, and a type I error rate of 0.05. Power for non-linear models calculated using a normal approximation. Supplementary material E. Deviations from SAP. Supplementary material F1. CONSORT flow diagram. Supplementary material F2. Characteristics of clusters and mothers by time period and intervention allocation. Supplementary material F3. Primary outcome results. Values are number and percentages unless stated otherwise. Supplementary material F4. Subgroup analysis results. Subgroup is location (rural/urban setting). Values are number and percentages unless stated otherwise. Supplementary material F5. Secondary outcome results. Values are number and percentages unless stated otherwise. Suppl [file 13063_2024_8328_MOESM1_ESM.docx]

# Supplementary material

Supplementary material A: Statistical Analysis Plan (SAP) Checklist v 1.0 2019

| Section/Item | Index | Description | Reported on page # |
| --- | --- | --- | --- |
| **Section 1: Administrative information** | | | |
| Trial and Trial registration | 1a | Descriptive title that matches the protocol, with SAP either as a forerunner or subtitle,  and trial acronym (if applicable) | 1 |
|  | 1b | Trial registration number | 3 |
| SAP Version | 2 | SAP version number with dates | 18 |
| Protocol Version | 3 | Reference to version of protocol being used | 18 |
| SAP revisions | 4a | SAP revision history | Supplementary material |
|  | 4b | Justification for each SAP revision | Supplementary material |
|  | 4c | Timing of SAP revisions in relation to interim analyses, etc. | Supplementary material |
| Roles and responsibility | 5 | Names, affiliations, and roles of SAP contributors | Supplementary material |
| Signatures of: | 6a | Person writing the SAP | Supplementary material |
|  | 6b | Senior statistician responsible | Supplementary material |
|  | 6c | Chief investigator/clinical lead | Supplementary material |
| **Section 2: Introduction** | | | |
| Background and rationale | 7 | Synopsis of trial background and rationale including a brief description of research question  and brief justification for undertaking the trial | 3-4 |
| Objectives | 8 | Description of specific objectives or hypotheses | 4 |
| **Section 3: Study Methods** | | | |
| Trial design | 9 | Brief description of trial design including type of trial (e.g., parallel group, multi-arm, crossover, factorial)  and allocation ratio and may include brief description of interventions | 5 |
| Randomization | 10 | Randomization details, e.g., whether any minimization or stratification occurred (including stratifying  factors used or the location of that information if it is not held within the SAP) | 6 |
| Sample size | 11 | Full sample size calculation or reference to sample size calculation in protocol  (instead of replication in SAP) | 8 |
| Framework | 12 | Superiority, equivalence, or noninferiority hypothesis testing framework, including which comparisons  will be presented on this basis | 5 |
| Statistical interim analysis and stopping guidance | 13a | Information on interim analyses specifying what interim analyses will be carried out  and listing of time points | 9 |
|  | 13b | Any planned adjustment of the significance level due to interim analysis | 9 |
|  | 13c | Details of guidelines for stopping the trial early | 9 |
| Timing of final analysis | 14 | Timing of final analysis, e.g., all outcomes analysed collectively or timing stratified  by planned length of follow-up | 9 |
| Timing of outcome assessments | 15 | Time points at which the outcomes are measured including visit “windows” | 7 |
| **Section 4: Statistical Principals** | | | |
| Confidence intervals and *P* values | 16 | Level of statistical significance | 10 |
|  | 17 | Description and rationale for any adjustment for multiplicity and, if so, detailing how the type 1 error  is to be controlled | 10 |
|  | 18 | Confidence intervals to be reported | 10 |
| Adherence and Protocol deviations | 19a | Definition of adherence to the intervention and how this is assessed including extent  of exposure | 15-16 |
|  | 19b | Description of how adherence to the intervention will be presented | 15-16 |
|  | 19c | Definition of protocol deviations for the trial | 10 |
|  | 19d | Description of which protocol deviations will be summarized | 10 |
| Analysis populations | 20 | Definition of analysis populations, e.g., intention to treat, per protocol,  complete case, safety | 10 |
| **Section 5: Trial Population** | | | |
| Screening data | 21 | Reporting of screening data (if collected) to describe representativeness  of trial sample | 5 |
| Eligibility | 22 | Summary of eligibility criteria | 5 |
| Recruitment | 23 | Information to be included in the CONSORT flow diagram | 10-11 |
| Withdrawal/ Follow-up | 24a | Level of withdrawal, e.g., from intervention and/or from follow-up | 10-11 |
|  | 24b | Timing of withdrawal/lost to follow-up data | 10-11 |
|  | 24c | Reasons and details of how withdrawal/lost to follow-up data will be presented | 10-11 |
| Baseline patient characteristics | 25a | List of baseline characteristics to be summarized | 11 |
|  | 25b | Details of how baseline characteristics will be descriptively summarized | 11 |
| **Section 6: Analysis** | | | |
| Outcome definitions |  | List and describe each primary and secondary outcome including details of: | 7 |
|  | 26a | Specification of outcomes and timings. If applicable include the order of importance of primary  or key secondary end points (e.g., order in which they will be tested) | 7 |
|  | 26b | Specific measurement and units (e.g., glucose control, hbA1c [mmol/mol or %]) | 7 |
|  | 26c | Any calculation or transformation used to derive the outcome (e.g., change from baseline, QoL score,  Time to event, logarithm, etc.) | 7 |
| Analysis methods | 27a | What analysis method will be used and how the treatment effects will be presented | 11-13 |
|  | 27b | Any adjustment for covariates | 14-15 |
|  | 27c | Methods used for assumptions to be checked for statistical methods | 17 |
|  | 27d | Details of alternative methods to be used if distributional assumptions do not hold, e.g., normality,  proportional hazards, etc. | 17 |
|  | 27e | Any planned sensitivity analyses for each outcome where applicable | 17 |
|  | 27f | Any planned subgroup analyses for each outcome including how subgroups are defined | 15 |
| Missing data | 28 | Reporting and assumptions/statistical methods to handle missing data (e.g., multiple imputation) | 17 |
| Additional analyses | 29 | Details of any additional statistical analyses required, e.g., complier-average causal effect10 analysis | 16-17 |
| Harms | 30 | Sufficient detail on summarizing safety data, e.g., information on severity, expectedness, and causality;  details of how adverse events are coded or categorized; how adverse event data will be analysed,  i.e., grade 3/4 only, incidence case analysis, intervention emergent analysis | 18 |
| Statistical software | 31 | Details of statistical packages to be used to carry out analyses | 18 |
| References | 32a | References to be provided for nonstandard statistical methods | N/A |
|  | 32b | Reference to Data Management Plan | N/A |
|  | 32c | Reference to the Trial Master File and Statistical Master File | N/A |
|  | 32d | Reference to other standard operating procedures or documents to be adhered to | N/A |

**Taken from the paper:** Gamble C, Krishan A, Stocken D, Lewis S, Juszczak E, Doré C, et al. Guidelines for the Content of Statistical Analysis Plans in Clinical Trials. JAMA. 2017;318(23):2337-43.

**Abbreviations:** CONSORT, Consolidated Standards of Reporting Trials; hbA1c, haemoglobin A1c; QoL, quality of life; SAP, statistical analysis plan.

For more information visit:

*The development of this checklist was funded by the* [*MRC Hubs for Trials Methodology Research*](https://www.methodologyhubs.mrc.ac.uk/)

Supplementary material B: Names, affiliations, and roles of SAP contributors.

**Statistical analysis plan**

| SAP Version Number | Protocol Version Number |
| --- | --- |
| *2.5* | *1.4* |

| Name of Author: | Laura Quinn | Role: | Trial Statistician  (Supporting the causal inference analysis) | Affiliation: | Institute of Applied Health Research  University of Birmingham |
| --- | --- | --- | --- | --- | --- |
| Signature of Author: | 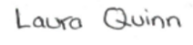 | Date: | 27/03/24 |  |  |
|  | James Martin |  | Trial Statistician (Supporting the main trial analysis) |  | Institute of Applied Health Research  University of Birmingham |
| Signature of Author: | 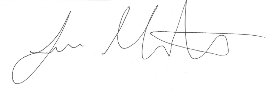 |  | 27/03/2024 |  |  |
|  | Sam Watson |  | Statistician (Leading the causal inference analysis) |  | Institute of Applied Health Research  University of Birmingham |
| Signature of  Author: | 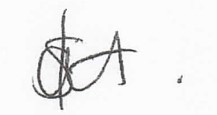 | Date | 27/03/2024 |  |  |
| Name of Chief Investigator: | Semira Manaseki-Holland | Role: | Chief Investigator | Affiliation: | Institute of Applied Health Research  University of Birmingham |
| Signature of Chief Investigator: | 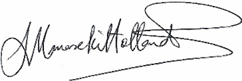 | Date: | Sent March  2024 for approval – signed May 14 2024 |  |  |
| **This Statistical Analysis Plan has been reviewed and approved by:** | | | | | |
| Name of Approver: | Karla Hemming | Role: | Senior Statistician (Leading the main trial analysis) | Affiliation: | Institute of Applied Health Research  University of Birmingham |
| Signature of Approver: | 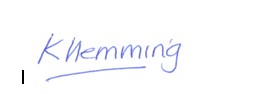 | Date: | 27/03/2024 |  |  |

Supplementary material C: List of additional secondary outcomes as outlined in protocol. These were termed “alternative outcomes” in the protocol. For each of the outcomes, the difference between the intervention and control group at 4 months post-intervention and 15 months post-intervention (primary assessment time) will be calculated.

| **Outcome category** | **Description** | **Method** | **Units** | **Covariates for adjustments** |
| --- | --- | --- | --- | --- |
| **Additional outcomes** | | | |  |
| Water and Food safety and hygiene behaviour | Availability and use of soap in key areas for handwashing (kitchen and toilet) | Observation | Dichotomous (for kitchen and toilet separately) | Age of the child  Mother’s educational status  Working mother at the time of the interview  Number of children <5 years in the household |
| ~~Food and water contamination~~ | *~~E. Coli~~* ~~count in family/adult food~~ | ~~Sample collection and field testing~~ | ~~cfu/g~~ | Due to budget cuts, this outcome is no longer being collected |
| Diarrhoea | 7-day parental report of child diarrhoea episodes (3 or more watery stools in 24hrs) | Survey | Dichotomous | Age of the child  Mother’s educational status  Working mother at the time of the interview  Number of children <5 years in the household |
|  | | | |  |
| ***Knowledge and behaviour*** | | | |  |
| Nutrition | Minimum dietary diversity of 5 out of 8 food groups: Breastmilk; Grains, roots and tubers; Legumes and nuts; Dairy products (infant formula, milk, yogurt, cheese); Flesh foods (meat, fish, poultry and liver/organ meats); Eggs; Vitamin A rich fruits and vegetables; Other fruits and vegetables | Survey | Dichotomous | Age of the child  Mother’s educational status  Working mother at the time of the interview  Number of children <5 years in the household |
| Nutrition frequency | A minimum meal frequency of: 2 or more solid or semi-solid or soft feeds for breastfeeding children age 6-8 months, or 3 or more solid or semi-solid or soft feeds for breastfeeding children age 9-23 months; or 4 or more solid or semi-solid or soft or milk feeds for non-breastfeeding children age 6-23 months where at least one of the feeds must be a solid, semi-solid, or soft feed. | Survey | Dichotomous | Age of the child  Mother’s educational status  Working mother at the time of the interview  Number of children <5 years in the household |
| Geophagy | Accessibility of play area to animals. | Observation | Dichotomous | Age of the child  Mother’s educational status  Working mother at the time of the interview  Number of children <5 years in the household |
| Maternal autonomy and uptake | Proportion of women who have achieved each stage of the mothers’ MaaCiwara intervention competition after pledging on the 4^th^ visit. | Intervention Log | Binary | NA |
| Maternal Knowledge | Knowledge score related to food-safety and hygiene | Survey | Proportion | Age of the child  Mother’s educational status  Working mother at the time of the interview  Marital status- 2^nd^ wife |
| ***Short-term microbiological and clinical outcomes*** | | | |  |
| Acute respiratory infection | In-patient hospitalisation (if given a bed to stay for observation, tests or treatment for >3 hours) for respiratory illness in past three months | Survey | Dichotomous | Age of the child  Mother’s educational status  Working mother at the time of the interview  Number of children <5 years in the household |
| **Long-term physiological outcomes** | | | |  |
| Physical growth | Mid-upper arm circumference (MUAC) (take average of three measures taken at each point in time) | On site measurement | Continuous | Age of the child  Mother’s educational status  Working mother at the time of the interview  Number of children <5 years in the household |
| **Social outcomes** | | | |  |
| Trust and solidarity survey measure | Reported levels of trust and solidarity in the community (WB measure) | Survey | Continuous | Mother’s education, mother working, Number of refugees/internal migrants in the community, wealth/revenue, age mother, ethnicity of mother, |
| Social cohesion and inclusion survey measure | The feeling of social cohesion and inclusion in the community (WB measure) | Survey | Continuous | Mother’s education, mother working, Number of refugees/internal migrants in the community,wealth/revenue age mother, ethnicity of mother, |

### Supplementary material D1: Power for outcomes in primary outcomes for combination of effect sizes and assuming an ICC of 0.02, a CAC of 0.8, and a type I error rate of 0.05. Power for non-continuous outcomes calculated using a normal approximation. pp = percentage point. For full details see main text. Of note the values in yellow are corrected from those published in the protocol.

| **Outcome** | **Assumed model** | **Assumed baseline** | **Effect size** | | **Obs. per cluster-period** | **Power** |
| --- | --- | --- | --- | --- | --- | --- |
| *Primary outcomes* | | | | | | |
| Water and Food safety and hygiene behaviour | Binomial-logistic | 50% | +5pp | | 27 mother-child pairs  4 opportunities per pair | 69% |
|  |  |  | +10pp | |  | >99% |
|  |  |  | +20pp | |  | >99% |
| Food and water contamination | Poisson | 10 cfu/g | -1 | | 10 samples per cluster | >99% |
|  |  |  | -2 | |  | >99% |
| Diarrhoea | Binomial-logistic | 13% | -2pp | | 27 mother-child pairs | 33% |
|  |  |  | -3pp | |  | 64% |
|  |  |  | -5pp | |  | 97% |
|  |  |  | -7pp | |  | >99% |
| **Outcome** | **Assumed model** | **Assumed baseline** | **Main effect** | **Interaction effect** | **Obs. per cluster-period** | **Power** |
| *Subgroup analyses* | | | | | | |
| Water and Food safety and hygiene behaviour | Binomial-logistic | 50% | 0pp | +5pp | 27 mother-child pairs  4 opportunities per pair | 23% |
|  |  |  | 0pp | +10pp |  | 68% |
|  |  |  | +5pp | +5pp |  | 23% |
|  |  |  | +5pp | +10pp |  | >70% |
| Food and water contamination | Poisson | 10 cfu/g | 0 | -1 | 10 samples per cluster | 29% |
|  |  |  | 0 | -2 |  | 84% |
|  |  |  | -2 | -1 |  | 34% |
|  |  |  | -2 | -2 |  | 91% |
| Diarrhoea | Binomial-logistic | 13% | 0pp | -2pp | 27 mother-child pairs | 11% |
|  |  |  | 0pp | -5pp |  | 54% |
|  |  |  | -2pp | -2pp |  | 13% |
|  |  |  | -2pp | -5pp |  | 65% |
|  |  |  | -5pp | -2pp |  | 18% |

### Supplementary material D2: Power for the three main outcomes for different combinations of effect sizes and assuming an ICC of 0.05, a CAC of 0.8, and a type I error rate of 0.05. Power for non-linear models calculated using a normal approximation.

| **Outcome** | **Assumed model** | **Assumed baseline** | **Effect size** | | **Obs. per cluster-period** | **Power** |
| --- | --- | --- | --- | --- | --- | --- |
| *Primary outcomes* | | | | | | |
| Water and Food safety and hygiene behaviour | Binomial-logistic | 50% | +5pp | | 27 mother-child pairs  4 opportunities per pair | 57% |
|  |  |  | +10pp | |  | >99% |
|  |  |  | +20pp | |  | >99% |
| Food and water contamination | Poisson | 10 cfu/g | -1 | | 10 samples per cluster | >99% |
|  |  |  | -2 | |  | >99% |
| Diarrhoea | Binomial-logistic | 13% | -2pp | | 27 mother-child pairs | 26% |
|  |  |  | -3pp | |  | 52% |
|  |  |  | -5pp | |  | 94% |
|  |  |  | -7pp | |  | >99% |
| **Outcome** | **Assumed model** | **Assumed baseline** | **Main effect** | **Interaction effect** | **Obs. per cluster-period** | **Power** |

### Supplementary material D3: Power for the three main outcomes for different combinations of effect sizes and assuming an ICC of 0.05, an CAC of 0.5, and a type I error rate of 0.05. Power for non-linear models calculated using a normal approximation.

| **Outcome** | **Assumed model** | **Assumed baseline** | **Effect size** | **Obs. per cluster-period** | **Power** |
| --- | --- | --- | --- | --- | --- |
| *Primary outcomes* | | | | | |
| Water and Food safety and hygiene behaviour | Binomial-logistic | 50% | +5pp | 27 mother-child pairs  4 opportunities per pair | 50% |
|  |  |  | +10pp |  | 98% |
|  |  |  | +20pp |  | >99% |
| Food and water contamination | Poisson | 10 cfu/g | -1 | 10 samples per cluster | >99% |
|  |  |  | -2 |  | >99% |
| Diarrhoea | Binomial-logistic | 13% | -2pp | 27 mother-child pairs | 23% |
|  |  |  | -3pp |  | 46% |
|  |  |  | -5pp |  | 89% |
|  |  |  | -7pp |  | >99% |

Supplementary material E: Deviations from SAP

This report follows the statistical analysis plan version (insert latest version) apart from the following deviations:

| **Deviation from SAP** | **Reason** |
| --- | --- |
|  |  |

### Supplementary material F1: CONSORT flow diagram

### Supplementary material F2: Characteristics of clusters and mothers by time period and intervention allocation

#### Characteristics of clusters by time period and intervention allocation. Number and percentage unless stated otherwise.

|  | **Baseline period** | | **4 months post-intervention** | | | **15 months post-intervention**  **(primary assessment)** | |
| --- | --- | --- | --- | --- | --- | --- | --- |
| **Cluster characteristic** | **Intervention**  **(N=)** | **Control**  **(N=)** | **Intervention**  **(N=)** | **Control**  **(N=)** | **Intervention**  **(N=)** | | **Control**  **(N=)** |
| **Location** |  |  |  |  |  | |  |
| Urban |  |  |  |  |  | |  |
| Rural |  |  |  |  |  | |  |
| **Households per cluster with children 0-36m**, *mean (SD)/median (IQR)* |  |  |  |  |  | |  |
| Have a functioning woman’s group  Have a school  Have a clinic/health centre  CLTS  Community-based Breastfeeding-friendly initiative/support programme  Water sources in the community:  Faucet  Pump/drill  Wells  Rainwater  Surface Water  Other |  |  |  |  |  | |  |

#### Characteristics of child, family and mothers by time period and intervention allocation. Number and percentage unless stated otherwise.

|  | **Baseline period** | | **4 months post-intervention** | | | **15 months post-intervention (primary assessment)** | |  |
| --- | --- | --- | --- | --- | --- | --- | --- | --- |
| **Characteristic** | **Intervention**  **(N=)** | **Control**  **(N=)** | | **Intervention**  **(N=)** | **Control**  **(N=)** | **Intervention**  **(N=)** | **Control**  **(N=)** | |
| Number of households in the yard, median (IQR)  Number of people in the household, median (IQR)  Number of children in household median (IQR)  Number children aged <6 in the household, median (IQR)  Number children aged 4 - 14 in the household, median (IQR)  Number people aged >14 in the household, median (IQR)  Mothers age, median (IQR)  Mothers Ethnicity  Bambara  Peul  Sénoufo  Sarakole  Malinké  Other  Mothers marital status  Never Married  Married to a husband who has only one wife  Married to a husband who has more than one wife  Widowed  Divorced  Separated  Number of children, median (IQR)  Number living children, median (IQR)  Number living children aged under 6 months, median (IQR)  Number living children aged 7 - 36 months, median (IQR)  Number living children aged 37 - 59 months, median (IQR)  Number living children aged over 5, median (IQR)  Did you attend a Koranic School  Mothers Education  No instruction  Started basic school but did not finish it  Completed basic school  Started high school, but did not finish it  Completed secondary school  Started high school, but didn't finish it  Has a graduate degree  Husband Education  No instruction  Started basic school but did not finish it  Completed basic school  Started high school, but did not finish it  Completed secondary school  Started high school, but didn't finish it  Has a graduate degree  Unknown  Mothers Reading Level  Cannot read at all  Can read parts of the sentence  Can read the whole sentence  No sentence in the language of the respondent  Grown crops  Have a vegetable garden  Water source for drinking (this season)  Faucet  Pump/drill  Wells  Rainwater  Surface Water  Other  Where is the water source for drinking (this season)  In the yard  Out of the yard |  |  | |  |  |  |  | |
|  |  |  | |  |  |  |  | |

Supplementary material F3: Primary outcome results. Values are number and percentages unless stated otherwise.

|  | **Baseline** | | **4 months post-intervention** | | | | | | **15 months post-intervention (primary assessment)** | | | | | | | |
| --- | --- | --- | --- | --- | --- | --- | --- | --- | --- | --- | --- | --- | --- | --- | --- | --- |
| **Primary outcomes** | **Intervention**  **(N=)** | **Control**  **(N=)** | **Intervention**  **(N=)** | **Control**  **(N=)** | **Relative effect size**  **(95% CI)**  **p-value*** | | **Absolute effect size**  **(95% CI)**  **p-value**** | | **Intervention**  **(N=)** | | **Control**  **(N=)** | | **Relative effect size**  **(95% CI)**  **p-value*** | | **Absolute effect size**  **(95% CI)**  **p-value**** | |
|  |  |  |  |  |  | |  | |  | |  | |  | |  | |
| **Behaviours met^1^** |  |  |  |  |  | |  | |  | |  | |  | |  | |
|  |  |  |  |  |  | |  | |  | |  | |  | |  | |
| **Food and water contamination^2^** |  |  |  |  | |  | |  | |  | |  | |  | |  |
|  |  |  |  |  | |  | |  | |  | |  | |  | |  |
| **Diarrhoea observed^3^** |  |  |  |  | |  | |  | |  | |  | |  | |  |
|  |  |  |  |  |  | |  | |  | |  | |  | |  | |

***** Relative effect sizes refer to odds ratios for behaviours met and diarrhoea outcomes, and rate ratios for the food and water contamination outcome

****** Absolute effects sizes refer to difference in the probabilities of the outcome, reported in percentage points

**^1^** Behaviours met refers to the proportion of water and food safety behaviours met out of all opportunities in an observation period

**^2^** Food and water contamination refers to the E.Coli count in a child’s food and water samples

**^3^** Diarrhoea refers to the observation of watery diarrhoea by data collectors and an history of at least two other such stools in the last 24 hours

Supplementary material F4: Subgroup analysis results. Subgroup is location (rural/urban setting). Values are number and percentages unless stated otherwise.

|  | **Baseline** | | **4 months post-intervention** | | | | **15 months post-intervention (primary assessment)** | | | | |
| --- | --- | --- | --- | --- | --- | --- | --- | --- | --- | --- | --- |
| **Primary outcomes by subgroup** | **Intervention**  **(N=)** | **Control**  **(N=)** | **Intervention**  **(N=)** | **Control**  **(N=)** | **Relative effect size**  **(95% CI)**  **p-value*** | **Absolute effect size**  **(95% CI)**  **p-value**** | **Intervention**  **(N=)** | **Control**  **(N=)** | **Relative effect size**  **(95% CI)**  **p-value*** | **Absolute effect size**  **(95% CI)**  **p-value**** | |
| **Behaviours met^1^** |  |  |  |  |  |  |  |  |  |  | |
| Rural |  |  |  |  |  |  |  |  |  |  | |
| Urban |  |  |  |  |  |  |  |  |  |  | |
| Difference*** | - | - | - | - |  |  | - | - |  |  | |
| **Food and water contamination^2^** |  |  |  |  |  |  |  |  |  |  | |
| Rural |  |  |  |  |  |  |  |  |  | |  |
| Urban |  |  |  |  |  |  |  |  |  | |  |
| Difference*** | - | - | - | - |  |  | - | - |  | |  |
| **Diarrhoea^3^** |  |  |  |  |  |  |  |  |  |  | |
| Rural |  |  |  |  |  |  |  |  |  |  | |
| Urban |  |  |  |  |  |  |  |  |  |  | |
| Difference*** | - | - | - | - |  |  | - | - |  |  | |

***** Relative effect sizes refer to odds ratios for behaviours met and diarrhoea outcomes, and rate ratios for the food and water contamination outcome

****** Absolute effects sizes refer to difference in the probabilities of the outcome, reported in percentage points

*** Difference refers to `ratio of ratios’ for relative measures and `difference of differences’ for absolute measures

**^1^** Behaviours met refers to the proportion of water and food safety behaviours met out of all opportunities in an observation period

**^2^** Food and water contamination refers to the E.Coli count in a child’s food and water samples

**^3^** Diarrhoea refers to the observation of watery diarrhoea by data collectors and an history of at least two other such stools in the last 24 hours

Supplementary material F5: Secondary outcome results. Values are number and percentages unless stated otherwise.

|  | **Baseline** | | | **4 months post-intervention** | | | | | | **15 months post-intervention (primary assessment)** | | | | | | | |
| --- | --- | --- | --- | --- | --- | --- | --- | --- | --- | --- | --- | --- | --- | --- | --- | --- | --- |
| **Secondary outcomes** | **Intervention**  **(N=)** | | **Control**  **(N=)** | **Intervention**  **(N=)** | **Control**  **(N=)** | **Relative effect size**  **(95% CI)**  **p-value** | | **Absolute effect size**  **(95% CI)**  **p-value** | | **Intervention**  **(N=)** | | **Control**  **(N=)** | | **Relative effect size**  **(95% CI)**  **p-value** | | **Absolute effect size**  **(95% CI)**  **p-value** | |
| **Knowledge and behaviour** |  | |  |  |  |  | |  | |  | |  | |  | |  | |
| Nutrition^1^ |  | |  |  |  |  | |  | |  | |  | |  | |  | |
| Geophagy^2^ |  | |  |  |  |  | |  | |  | |  | |  | |  | |
| Maternal autonomy^3^ |  | |  |  |  | |  | |  | |  | |  | |  | |  |
| **Short-term microbiological and clinical** |  | |  |  |  | |  | |  | |  | |  | |  | |  |
| Acute respiratory infection^4^ |  | |  |  |  | |  | |  | |  | |  | |  | |  |
| Diarrhoea hospitalisation_5_ |  | |  |  |  | |  | |  | |  | |  | |  | |  |
| Enteric infection^6^ |  | |  |  |  | |  | |  | |  | |  | |  | |  |
| ETEC | |  |  |  |  | |  | |  | |  | |  | |  | |  |
| EAEC | |  |  |  |  | |  | |  | |  | |  | |  | |  |
| EPEC | |  |  |  |  | |  | |  | |  | |  | |  | |  |
| Astrovirus | |  |  |  |  | |  | |  | |  | |  | |  | |  |
| Sapovirus | |  |  |  |  | |  | |  | |  | |  | |  | |  |
| Rotavirus | |  |  |  |  | |  | |  | |  | |  | |  | |  |
| Adenovirus | |  |  |  |  | |  | |  | |  | |  | |  | |  |
| Giardia | |  |  |  |  | |  | |  | |  | |  | |  | |  |
| Crytosporidium | |  |  |  |  | |  | |  | |  | |  | |  | |  |
| E.Histolytica | |  |  |  |  | |  | |  | |  | |  | |  | |  |
| **Long-term physiological outcomes** |  | |  |  |  | |  | |  | |  | |  | |  | |  |
| Weight^7^ |  | |  |  |  | |  | |  | |  | |  | |  | |  |
| Height^8^ |  | |  |  |  | |  | |  | |  | |  | |  | |  |
| Cognitive development^9^ |  | |  |  |  |  | |  | |  | |  | |  | |  | |

***** Relative effect sizes refer to odds ratios for nutrition, geophagy, maternal autonomy, acute respiratory infection, diarrhoea hospitalisation, enteric infection outcomes, and rate ratios for the cognitive development outcome

****** Absolute effects sizes refer to difference in the probabilities of the outcome, reported in percentage points except for weight and height outcomes where mean differences are reported

**^1^** Nutrition refers to the minimum acceptable diet based on dietary diversity and meal frequency fed during the day

**^2^** Geography refers to the number of behaviours observed out of total opportunities in the assessment of child play environment and behaviour in a period

**^3^** Maternal autonomy refers to the number of decisions women participate in based on women’s autonomy measure from DHS survey

^4^ Acute respiratory infection refers to a parental report of cough and difficult breathing in the last seven days

^5^ Diarrhoea hospitalisation refers to in patient hospitalisation for diarrheal disease in the past three months

^6^ Enteric infection refers to a qualitative PCR of pathogens

^7^ Weight refers to weight for age based on WHO international growth tables

^8^ Height refers to height for age based on WHO international growth tables

^9^ Cognitive development refers to ASQ3 score for age

Supplementary material F6: Additional analysis results, part 1

#### Directed acyclic graph (DAG) representing casual assumptions between intervention and three primary outcomes.

Diarrhoea

Intervention

Water, food safety and hygiene behaviour

Food and water contamination

#### Mediation effect of water, food safety and hygiene behaviour and water contamination on the association between the intervention and diarrhoea. Direct, indirect and total effects reported with 95% confidence intervals. Proportion of total effect mediated also reported.

| **Effect of intervention on diarrhoea via:** | **Direct effects (95% CI)** | **Indirect effects (95% CI)** | **Total effects (95% CI)** | **Proportion of total effect mediated** |
| --- | --- | --- | --- | --- |
| Water, food safety and hygiene behaviour |  |  |  |  |
| Food and water contamination |  |  |  |  |

### Supplementary material F7: Additional analysis results, part 2

#### Causal diagram is represented as a directed acyclic graph (DAG) displaying the causal assumptions of the study.

Growth

Development

Water, food safety and hygiene behaviour

Food and water contamination

Enteric infection

Diarrhoea

Nutrition

Geophagy

Maternal autonomy

Intervention

#### Direct and indirect effects for casual assumption of the study. Estimates are reported with 95% confidence intervals and p-values.

|  | **Estimate (95% CI)** | **p-value** |
| --- | --- | --- |
| **Direct effects** |  |  |
| Intervention → Diarrhoea |  |  |
| Intervention → Geophagy |  |  |
| … |  |  |
| Enteric infection → Diarrhoea |  |  |
|  |  |  |
| **Indirect effects** |  |  |
| Intervention → Maternal autonomy → Water, food safety and hygiene behaviour |  |  |
| …. |  |  |
| Intervention → Maternal autonomy → Geophagy → Enteric infection → Diarrhoea |  |  |
| Intervention → Maternal autonomy → Water, food safety and hygiene behaviour → Food and water contamination → Enteric infection → Diarrhoea |  |  |
|  |  |  |

Supplementary material G: Statistical Analysis Plan (SAP) Amendments

| **SAP version number** | **SAP section number** | **Description of and reason for change** | **Timing of change with respect to interim analysis/ final analysis/ database lock** | **Blind Reviewer**  (if blind review not required put N/A in name row) | |
| --- | --- | --- | --- | --- | --- |
| 2.1 |  | Amendments in response to DMC comments round 1 | Before database lock  After baseline analysis for data quality monitoring | Name: |  |
|  |  |  |  | Signature: |  |
|  |  |  |  | Date: |  |
| *2.2* |  | Amendments in response to DMC comments round 2 | Before database lock  After baseline analysis for data quality monitoring |  |  |
| *2.2* |  | Updated roles:  LQ and SW to work on mediation analysis only  KH and JM to work on main trial analysis | Before database lock  After baseline analysis for data quality monitoring |  |  |
| *2.3* | C1 | Correction to the power calculation as published in protocol | Before database lock  After baseline analysis for data quality monitoring |  |  |
| *2.4* | Table 1 | Definition of primary outcome – more detail of exact definition. This was pre-planned as per statement in protocol about details being reviewed when intervention design agreed.    Change to planned control condition (note this was communicated by SM but does not appear as a change in version 1.4 of protocol) | Before database lock  After baseline covariate analysis for data quality monitoring | May 2024 |  |
| *2.4* | Table 2 | For the outcomes: weight, height and arm circumference there were three measures in the dataset at each point in time. Amendment to clarify take average of those available.  Weight appears twice in the list of outcomes – remove the second occurrence (Appendix B) | Before database lock  After baseline covariate analysis for data quality monitoring | May 2024 |  |
| *2.4* | Section 7.1 | Primary Outcome “E. Coli count in child’s food and water samples” (Table 1):  The distribution of this variable is highly skewed with very extreme values. Change to analysis plan to explore appropriate transformations or analyse in a dichotomous format (O vs >0). | Before database lock  After baseline covariate analysis for data quality monitoring | May 2024 |  |
| *2.4* | Section 7.1 | Clarification to section 7 that generally components of composites will be analysed as exploratory outcomes. | Before database lock  After baseline covariate analysis for data quality monitoring | May 2024 |  |
| *2.4* | Table 2 | Outcome: Minimum acceptable diet based on minimum dietary diversity **and** minimum meal frequency they are fed during the day (DHS survey, see below) Table 2  Add the cut point for definition of acceptable (this was copied from Appendix B – as already defined in a different part of the SAP). | Before database lock  After baseline covariate analysis for data quality monitoring | May 2024 |  |
| *2.4* | Table 2 | Outcome Geophagy (Table 2). Issues and proposal:  Add clear definition is required.  “These include:  1.Observed no geophagy,  2.Observed supervision during play,  3.Observed sitting/playing on a clean surface.” | Before database lock  After baseline covariate analysis for data quality monitoring | May 2024 |  |
|  | Table 2 | Outcome Enteric infection (Table 2). Many of these infections are observed very infrequently and there are many of these.  Change to composite rather than report each separate infection | Before database lock  After baseline covariate analysis for data quality monitoring | May 2024 |  |
